# Prevalence and Phage-Based Biocontrol of Methicillin-Resistant *Staphylococcus aureus* Isolated from Raw Milk of Cows with Subclinical Mastitis in Vietnam

**DOI:** 10.3390/antibiotics13070638

**Published:** 2024-07-10

**Authors:** Hoang Minh Son, Hoang Minh Duc

**Affiliations:** 1 Department of Anatomy and Histology, Faculty of Veterinary Medicine, Vietnam National University of Agriculture, Trau Quy, Gia Lam, Hanoi 12400, Vietnam; hson@vnua.edu.vn; 2 Laboratory of Veterinary Microbiology, Center of Research Excellence and Innovation, Vietnam National University of Agriculture, Trau Quy, Gia Lam, Hanoi 12400, Vietnam; 3 Department of Veterinary Public Health, Faculty of Veterinary Medicine, Vietnam National University of Agriculture, Trau Quy, Gia Lam, Hanoi 12400, Vietnam

**Keywords:** *Staphylococcus aureus*, MRSA, phage, biocontrol

## Abstract

*S. aureus*, particularly methicillin-resistant *S. aureus*, has been recognized as a main cause of bovine mastitis and food poisoning. This study investigated the prevalence, antibiotic resistance, and phage-based biocontrol of *S. aureus* and methicillin-resistant *S. aureus* isolated from raw milk of cows with subclinical mastitis. The results showed that the prevalence of *S. aureus* and methicillin-resistant *S. aureus* was 12% (48/400) and 1.5% (6/400), respectively. The *S. aureus* isolates were highly resistant to penicillin (72.92%), erythromycin (43.75%), and tetracycline (39.58%). Out of 48 *S. aureus* isolates, 6 were identified as methicillin-resistant strains. Among them, one isolate was found to harbor the *sea* gene. A total of 5 phages were recovered from 50 pork and 50 chicken meat samples, 1 from pork and 4 from chicken meat samples. Phage PSA2 capable of lysing all 6 methicillin-resistant isolates was selected for characterization. The use of phage PSA2 completely inactivated methicillin-resistant *S. aureus* SA33 in raw milk at both 24 °C and 4 °C, indicating its potential as a promising antibacterial agent in controlling methicillin-resistant *S. aureus* in raw milk and treating bovine mastitis.

## 1. Introduction

Mastitis has been known to be one of the most common and costly diseases in the dairy industry worldwide [1]. In the United States (US), this disease affects approximately one-third of all dairy cows, resulting in an annual economic loss to the dairy industry of over 2 billion dollars [2]. In the world, around 40% of cows are infected with mastitis, costing EUR 125 billion due to reduced milk production, discarded milk, early culling, veterinary services, and labor costs [3,4]. Mastitis is mainly caused by bacterial infection. Depending on the pathogenicity of bacteria and host immunity, mastitis can occur in clinical (CM) and subclinical (SCM) forms [5]. Dairy cows infected with CM usually show the following obvious symptoms: inflamed udder, lumps, fever, fatigue, loss of appetite, and clots in milk. On the other hand, dairy cows infected with SCM exhibit invisible symptoms in the udder and milk [6]. Counting somatic cells in milk is the most commonly used diagnostic method for detecting the subclinical form of mastitis, and as a result, a few farms in Vietnam regularly conduct this test before selling their raw milk [7]. Recently, SCM has been detected 15–50 times more frequently in dairy cows than CM [7]. In addition, SCM leads to greater economic losses than CM due to challenges in diagnosis, longer persistence, and silent spread in dairy farms [7].

*S. aureus* has been recognized as a major cause of bovine mastitis, causing 40% of mastitis cases in many countries around the world [8,9]. Therefore, this pathogen can easily enter milk from the teats and udders of cows infected with mastitis during improper milking process [10]. In an ideal medium containing necessary nutrients, such as raw milk, *S. aureus* can multiply to reach a high cell concentration that enables it to produce staphylococcal enterotoxins (SEs) [11]. To date, about 20 different SEs have been identified, with the most common classical SEs being SEA, SEB, SEC, SED, and SEE, which are responsible for 95% of staphylococcal food poisoning (SFP). Although pasteurization can eliminate *S. aureus* cells in raw milk, the SEs retain their biological activity even after pasteurization since they are heat-stable toxins [12]. Moreover, raw milk is increasingly consumed worldwide as raw milk is believed to contain more amino acids, antimicrobials, vitamins, minerals, and fatty acids than pasteurized milk [13,14]. In the US, 30 states have recently approved the consumption of raw milk, and about 3% of the US population consumes raw milk [15,16]. As a result, contaminated milk and dairy products have been considered important vehicles for transmitting *S. aureus* from dairy cows infected with mastitis to humans [17]. 

The emergence of antibiotic-resistant *S. aureus* (ARS) strains in milk products has been considered a significant challenge for the dairy industry and food safety, as it has led to limited therapeutic options in both human and veterinary medicine [3,18]. The abuse and misuse of antibiotics in the dairy industry for animal disease prevention, treatment, and growth promotion have been attributed to the rise of antimicrobial resistance (AMR), including ARS [19,20]. The most important ARS variant has been recognized as methicillin-resistant *S. aureus* (MRSA) which is also known as “superbug” or “resistant staph”. In 2017, MRSA was classified by the World Health Organization (WHO) into a high-priority bacterial group for research and development of new antibiotics. Recently, the occurrence of MRSA in raw milk has been increasingly reported worldwide, indicating the potential risk for the transmission of MRSA to humans through the consumption of raw milk and raw milk products, thereby raising public health concerns [21,22,23,24].

Bacteriophages, also known as bacterial viruses, have recently emerged as a promising tool for controlling foodborne pathogens in various types of foods, including raw milk [25,26,27,28]. They possess the unique ability to infect and lyse specific bacterial hosts without disturbing gut microbiota. On the other hand, phages do not have the mechanism to infect human and animal cells [29]. In addition, bacterial viruses have self-replication capabilities, ensuring their sustainable presence and antibacterial efficacy with a single dose [30]. Moreover, bacteriophages can kill antibiotic-resistant bacteria, offering a potential alternative to address the global issue of antibiotic resistance [31]. Furthermore, many phages contain polysaccharide depolymerizers, capable of degrading the extracellular polymeric substance, a significant structural component of most bacterial biofilms [32].

So far, there is limited information about the prevalence and antimicrobial resistance profile of *S. aureus* and MRSA originating from raw milk of cows with subclinical mastitis (SCM) in Vietnam. Therefore, this study aimed to (1) determine the prevalence and antibiotic resistance profile of *S. aureus* and MRSA isolated from raw milk of cows with SCM in Hanoi, Vietnam; and (2) isolate phages capable of controlling MRSA in raw milk.

## 2. Results

### 2.1. Prevalence of S. aureus in Raw Milk

In this study, *S. aureus* was found in 48 (12%) out of 400 raw milk samples tested. Presumptive *S. aureus* isolates, confirmed by Gram-staining, coagulase test, and *nuc* gene, were preserved at −86 °C for further use. To avoid duplication, only one isolate from each positive milk sample was randomly selected for the antimicrobial susceptibility test.

### 2.2. Antimicrobial Susceptibility Profile of S. aureus Isolates

The results of the antimicrobial susceptibility test are presented in Table 1. *S. aureus* isolates showed the highest resistance rates to penicillin (72.92%), followed by erythromycin (43.75%), and tetracycline (39.58%). In contrast, *S. aureus* isolates exhibited the lowest resistance rates to cefoxitin (12.5%), ciprofloxacin (16.67%), clindamycin (18.75%), and chloramphenicol (20.83%).

The results in Table 2 reveal that 95.83% (46/48) of *S. aureus* isolates were resistant to at least one antibiotic tested and exhibited 24 resistance patterns. The most common resistance phenotypes were PEN and PEN-TET, counting for 27.08% (13/48) and 8.33% (4/48) of the isolates, respectively. Out of 48 *S. aureus* isolates, 37 (77.08%) showed resistance to 1 to 5 antibiotics, while the resistance rate to 6 to 9 antibiotics was found to be 18.75% (9/48). Table 2 also shows that 17 (35.42%) out of 48 *S. aureus* isolates were identified as multidrug-resistant strains.

### 2.3. Molecular Characterization of Methicillin-Resistant S. aureus Isolates

Results of multiplex PCR indicated that all presumptive MRSA isolates were positive for *spa* and *mecA* genes, confirming that they were MRSA strains. None of the MRSA isolates carried *mecC* and *pvl* gene. The findings also showed that only 1 out of 6 MRSA isolates carried the enterotoxin gene (*sea* gene). This isolate (SA33) was selected as a bacterial host for phage isolation (Table 3).

### 2.4. Isolation of Bacteriophages against MRSA

**Figure 1:**
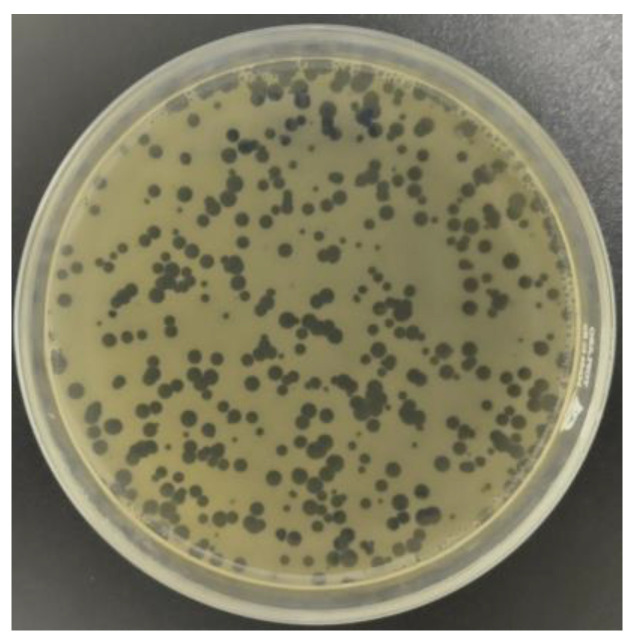
Large and clear plaques of phage PSA2 on MRSA SA33.

A total of 5 phages were isolated from 100 meat samples, of which 1 phage was isolated from pork and 4 recovered from chicken meat samples, suggesting that chicken meat was a good source for phage isolation. The isolated phages were designated as PSA1, PSA2, PSA3, PSA4, and PSA5, respectively. All isolated phages were able to produce clear and large plaques and easily propagated to reach a high titer of 10^10^ PFU/mL (Figure 1).

### 2.5. Phage Characterization

The lytic spectrum of 5 isolated phages on 6 MRSA isolates is shown in Table 4. Four phages of chicken meat origin, PSA1, PSA2, PSA3, and PSA4, lysed 6 (100%) out of 6 MRSA isolates tested. Phage PSA5, isolated from pork and exhibiting a narrower host range, lysed 5 (83.33%) out of 6 MRSA isolates. Since phage PSA2 produced clear plaques on all 6 MRSA isolates, it was selected for further characterization.

**Figure 2:**
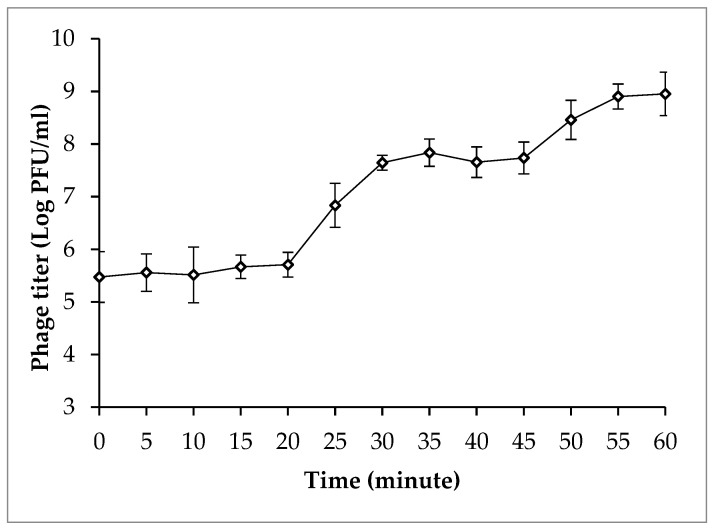
One-step growth curve of phage PSA2 in SA33. Error bars show standard deviations.

A one-step growth curve in Figure 2 reveals that phage PSA2 had a relatively short latent period of 20 min and a large burst size of 138 PFU/cell in SA33.

**Figure 3:**
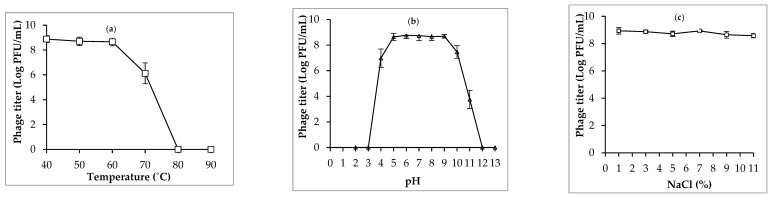
Effects of temperature (**a**), pH (**b**) and NaCl (**c**) on the stability of phage PSA2. Error bars show standard deviations.

The stability of phage PSA2 is detailed in Figure 3. Overall, phage PSA2 showed high heat tolerance at temperatures from 40 °C to 60 °C. However, the thermal stability of phage PSA2 was significantly reduced at 70 °C. At temperatures over 80 °C, infectious phage particles were not detected after 30 min of exposure. Phage PSA2 also had a good pH stability at pH from 5 to 9, but the phage was completely inactivated at pH ≤ 3 and pH ≥ 12. Similarly, phage PSA2 exhibited great NaCl stability; no significant reduction in phage titer was observed after 60 min of treatment at NaCl concentration from 1% to 11%.

**Figure 4:**
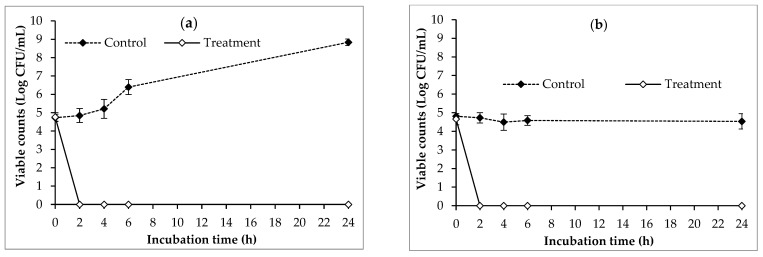
Effect of phage PSA2 on the viability of SA33 in raw milk stored at 24 °C (**a**) and 4 °C (**b**). SA33 was inoculated in 5 mL of raw milk at a final concentration of 10^5^ CFU/mL without (dashed line) and with phage PSA2 at 10^9^ PFU/mL (solid line). Error bars show standard deviations.

Figure 4 shows the efficacy of phage PSA2 in reducing viable counts of SA33 in raw milk at 24 °C and 4 °C. In the experiment carried out at 24 °C, viable counts of SA33 in the control group increased gradually and reached 8.84 log after 24 h of incubation. Conversely, the application of phage PSA2 rapidly decreased the bacterial host counts in the treatment group to under detection limit (<10^2^ CFU/mL) after 2 h of incubation and the regrowth was not observed at 24 h (Figure 4a). When stored at 4 °C, viable counts of SA33 in control group were maintained at a level equal to the initial inoculum. On the other hand, viable counts of SA33 in the treatment group were reduced to be under the detection limit after 2 h of phage treatment (Figure 4b). 

## 3. Discussion

In the current study, 48 (12%) out of 400 raw milk samples were positive for *S. aureus*. These rates are in line with a previous study in Nepal, which reported that 15.2% (29/191) of CMT-positive milk was contaminated with *S. aureus* [33]. However, the prevalence of *S. aureus* in our study is higher than in a study conducted by Kiraly et al. (2024) in Slovakia, in which 6 (2.8%) out of 215 milk samples from cows with SCM were found to be contaminated with *S. aureus* [34]. Conversely, several studies have reported a higher prevalence of *S. aureus* in raw milk. When investigating the antibiotic resistance of *S. aureus* isolated from raw milk of cows with SCM in Pakistan, Haq et al. (2024) found that 30.32% (94/310) of milk samples were positive for *S. aureus* and 11 out of 94 *S. aureus* isolates were identified as MRSA. Another study conducted in China showed that the incidence of *S. aureus* in bovine mastitis milk was 24.8% (31/125) [35]. In addition, Wang et al. (2018) found that the detection rate of *S. aureus* in raw milk from cows with mastitis in China was 46.2% (90/195) [36]. A higher prevalence of *S. aureus* in SCM milk samples was reported by Ren et al. (2020), *S. aureus* was found in 77.38% of milk samples [37]. The variation in the prevalence of *S*. *aureus* in the previous studies and this study may be due to the differences in sample characteristics (size, season, type), isolation method, and geographic locations.

Antimicrobial resistance has been recognized as one of the most serious threats to global public health [38]. The excessive use and misuse of antibiotics in food-producing animals are the main factors contributing to the development of AMR and the spread of antibiotic-resistant bacteria worldwide [39]. Antibiotics have been employed as feed additives to control disease and promote animal growth for almost 70 years [40]. It was estimated that 11,000 tons of antibiotics were used for food-producing animals in 2019. The antibiotics applied for disease prevention and promoting animal growth in Africa, the European Union, and the United States accounted for approximately 50–80% of the total antibiotics used in animals [39,41]. The use of antibiotics in food-producing animals is expected to rise by 11.5% (up to 200,235 tons) in 2030 [42]. In our study, *S. aureus* isolates exhibited high resistance rates to penicillin, erythromycin, and tetracycline, with 35.42% (17/48) of them being MDR strains. Similar results were found by Luna et al. (2023), who reported that 100% and 72.72% of *S. aureus* strains isolated from milk in Pakistan were resistant to penicillin and tetracycline, respectively. In addition, 20% of the isolates in Pakistan were determined as MDR [3]. The high resistance rates of *S. aureus* isolates to penicillin (96.3%) and tetracycline (98.1%) were also observed in a study performed by Gao et al. (2011) in China [43]. Likewise, Ren et al. (2020) found that 58.5% and 44.6% of *S. aureus* isolated from mastitis milk were resistant to penicillin and erythromycin, respectively. In the dairy industry, mastitis and lameness are the most common diseases. Beta-lactams and macrolides are frequently applied to treat bovine mastitis [44,45], while the most common antibiotics used for lameness are tetracycline, cephalosporin, and non-cephalosporin beta-lactams [20]. Dairy farms also use antibiotics for disease prevention. A previous survey showed that over 90% of dairy farms employed antibiotic dry cow therapy and administered intramammary antibiotics after the final milking of lactation. Approximately 80% of farms applied antibiotic dry cow therapy to treat all cows on the farm. The most common antibiotics used for dry cow therapy were penicillin G/dihydrostreptomycin and cephapirin [20]. This partly explains the high resistance rates of *S. aureus* isolates to penicillin, erythromycin, and tetracycline observed in this study.

Enterotoxigenic MRSA poses a significant challenge in both animal and human healthcare due to its resistance to multiple antibiotics and ability to produce enterotoxins that lead to food poisoning. In our study, 6 (12.5%) out of 48 *S. aureus* isolates were confirmed as MRSA through the detection of the *mecA* gene, indicating the prevalence of MRSA in raw milk samples was 1.5% (6/400). This percentage is higher than the findings of a study in Nepal, reporting that 1.05% (2/191) of CM milk was positive for MRSA [33]. In contrast, a study conducted in Indonesia revealed a higher detection rate of MRSA, with 9 (10.5%) MRSA strains recovered from 86 SCM milk samples [46]. The results of the Iran survey also showed that 3.88% of SCM milk contained MRSA [47]. All MRSA isolates in this study were classified as MDR strains, showing resistance to 6 to 10 antibiotics. Compared to methicillin-susceptible isolates, MRSA strains exhibited higher levels of antibiotic resistance. One of the explanations for this phenomenon could be that MRSA strains have a greater capacity to acquire mobile genetic elements as transposons or integrated plasmids carrying AMR genes than methicillin-susceptible strains [48]. The possibility cannot be excluded that multidrug-resistant MRSA isolates in this study may be strains of human origin, that contaminated milk during the milking process [49]. Heat-stable enterotoxins are the most notable virulence factors responsible for food poisoning, toxic shock-like syndromes, and allergic and autoimmune diseases [50]. In this study, only one out of six MRSA isolates was found to carry the *sea* gene, consistent with the previous study that reported 52.9% (9/17) of *S. aureus* isolates from mastitis cases on 13 New York farms were positive for the *sea* gene [51].

Phage-based biocontrol of foodborne pathogens has gained increasing attention from both researchers and the food industry, particularly the dairy sector, due to its high safety, cost-effectiveness, and growing demands of customers for food without chemical preservatives [25,28]. Various commercial phage products such as ListShield^TM^, Listex P-100^TM^, EcoShield^TM^, SalmoFresh^TM^, and Salmonelex^TM^ (Intralytix, Robert Fulton Drive, Colombia, MD, USA) have received official approval from the United States Food and Drug Administration (FDA) and the United States Department of Agriculture (USDA) as food additives to control unwanted bacteria in foods [52,53]. Our previous studies conducted in Japan have shown the efficacy of phage SA46-CTH2 in controlling methicillin-susceptible *S. aureus* (MSSA) in pasteurized milk [25]. This study aimed to evaluate the effectiveness of phage PSA2 newly isolated from Vietnamese chicken meat in controlling enterotoxigenic multidrug-resistant MRSA strain recovered from raw milk of cows with subclinical mastitis in Vietnam. Our data indicates that phage PSA2 rapidly decreased the viable counts of the bacterial host in the treatment group to below the detection limit after 2 h of incubation at 24 °C and 4 °C, resulting in an over 3 log CFU/mL reduction compared to the untreated control group. To the best of our knowledge, few studies have explored the lytic capacity of phages in controlling MRSA in raw milk, with most studies focusing on the effect of phages on the viability of MSSA in heat-treated milk. Mohammadian et al. investigated the efficacy of phages against MDR and methicillin-resistant *S. aureus* strains isolated from bovine mastitis in high-ultra temperature (HUT) milk [54]. After 6 of storage at 37 °C, the lytic activity of phages against MDR *S. aureus* in HUT milk was not observed. However, the phages reduced bacterial host by approximately 3 log after 8 h of treatment compared to a control without phage addition [54]. In the present study, the phage PSA2 formed large and clear plaques on MRSA isolates. Additionally, the phage also exhibited a short latent period of 20 min and a large burst size of 138 PFU/cell in SA33. These findings indicated the strong lytic infectivity of phage PSA2 and partly elucidated the rapid inactivation of SA33 by phage PSA2 observed in our study. Temperature has been recognized as one of the most common extrinsic factors influencing the outcome of phage application [55]. It has been documented that higher temperature leads to better phage efficacy, and the lytic activity of phages may diminish or disappear at refrigeration temperatures [56,57]. Phage PSA2 significantly reduced viable counts of SA33 at both room temperature (24 °C) and refrigeration temperature (4 °C), demonstrating its potential as a natural milk preservative for controlling MRSA under real conditions. Moreover, the strong lytic activity of phage PAS2 in milk at various temperatures also indicates its potential as an alternative to antibiotics to treat bovine mastitis.

## 4. Materials and Methods

### 4.1. Isolation of S. aureus from Raw Milk

Raw milk samples (400) positive for the California mastitis test (CMT) were randomly collected from dairy farms in Hanoi, Vietnam. Samples were kept in an icebox and immediately transported to the Laboratory of Veterinary Public Health, Vietnam National University of Agriculture. *S. aureus* was isolated according to the plate count method APHA 39.63:2015 [58]. In short, the sample was serially diluted in buffered peptone water (BPW, Oxoid, ThermoFisher, Hants, UK). Appropriate dilutions (100 μL) were then plated on Baird Parker agar (PB, Oxoid, ThermoFisher, Hants, UK) supplemented with egg yolk tellurite and incubated at 37 °C for 24–48 h. After the incubation, typical colonies of *S. aureus*, black or gray in color and surrounded by an opaque halo, were picked up for Gram-staining and cultured in Brain Heart Infusion (BHI, Oxoid, ThermoFisher, Hants, UK) broth overnight at 37 °C for coagulase testing. Presumptive *S. aureus* strains were confirmed by PCR detection of the thermonuclease gene (*nuc*) before being stored at −86 °C for further experiments [59].

### 4.2. Antimicrobial Susceptibility Profile of S. aureus Isolates

Antibiotic susceptibility testing of *S. aureus* isolates was carried out by the Kirby–Bauer disk diffusion method on Mueller–Hinton agar (MHA, Oxoid, ThermoFisher, Hants, UK). Initially, 4–5 colonies of *S. aureus* on Tryptone Soy agar (TSA, Oxoid, ThermoFisher, Hants, UK) were mixed with 2 mL of sterile saline (0.85%) in Eppendorf tubes to obtain the turbidity equal to 0.5 McFarland standards, corresponding to 10^8^ CFU/mL. Subsequently, the bacterial suspension was spread on the MHA surface using a sterile swab and left for 3–5 min at room temperature. Following this, antibiotic disks were placed on the surface of the plate and incubated at 37 °C for 16–24 h. Antibiotics used in this study consisted of the following: penicillin, cefoxitin, gentamicin, tetracycline, chloramphenicol, erythromycin, clindamycin, ciprofloxacin, and trimethoprim/sulfamethoxazole (Oxoid, ThermoFisher, Hants, UK). After the incubation, the diameters of the inhibition zones were assessed and interpreted according to the guidelines of the Clinical and Laboratory Standard Institute (CLSI) [60]. The tested *S. aureus* isolates resistant to at least three or more antibiotic classes were identified as multidrug-resistant strains.

### 4.3. Molecular Characterization of Methicillin-Resistant S. aureus Isolates

Six *S. aureus* isolates resistant to cefoxitin were identified as presumptive MRSA strains. These isolates were selected for molecular characterization. The DNA of *S. aureus* isolates was extracted by GeneJet Genomic DNA purification kit (Thermoscientific, Vilnius, Lithuania). The *mecA, mecC, spa, pvl,* and enterotoxin genes were detected using PCR according to the previously described methods [61]. The *S. aureus* M17 from our bacterial collection that was previously isolated from raw milk and positive for *mecA, spa,* and *pvl* was used as a positive control. Primers used in this study are shown in Table 5.

### 4.4. Isolation of Bacteriophages against Methicillin-Resistant S. aureus Isolates

Meat samples (50 pork and 50 chicken meat) were purchased from local supermarkets in Hanoi, Vietnam for isolating phages specific to multidrug-resistant MRSA SA33 carrying the *sea* gene according to the protocol described by Duc et al. (2020) [27]. Briefly, a 25 g sample of meat was homogenized with 100 mL of Luria Bertani broth (LB, Oxoid, ThermoFisher, Hants, UK) supplemented with 10 mM CaCl_2_ and 100 µL of overnight culture of SA33 and incubated overnight at 37 °C with shaking. Following the enrichment, a proportion of the sample (10 mL) was collected and centrifuged at 12,000× *g* for 5 min at 4 °C to obtain the supernatant, which then passed through a 0.45 µm pore size membrane filter (Merck Millipore, Ireland). Afterward, the filtrate was serially diluted in saline magnesium (SM) buffer (0.05 M Tris-HCl buffer, pH 7.5, containing 0.1 M NaCl, 0.008 M MgSO4, and 0.01% gelatin) and mixed with 100 µL of overnight host culture and 4 mL of molten top agar [LB broth with 0.4% (*w*/*v*) agar] before being poured on TSA. The double-layer agar plates were incubated overnight at 37 °C. After incubation, a single plaque was chosen and resuspended in SM buffer to produce plaque suspension. For phage purification, the plaque suspension was serially diluted in SM buffer, mixed with host culture and molten top agar, and then poured onto TSA to generate well-separated plaques. This purification step was repeated for at least three rounds. Finally, the phage was propagated to achieve a high titer (>10^9^ PFU/mL) before it was preserved at 4 °C for further use.

### 4.5. Characterization of Isolated Phages

#### 4.5.1. Host Range of Isolated Phages

The infectivity of 5 isolated phages was examined on 6 isolated MRSA strains by a spot test [63]. Briefly, 10 µL of phage suspension was dropped on the surface of the double-layer agar inoculated with a bacterial host and incubated overnight at 37 °C. The following day, the presence of clear plaque indicated the lysis of the bacterial host by phages. Conversely, the absence of clear plaque was regarded as indicating no lysis.

#### 4.5.2. One-Step Growth Curve of Isolated Phage PSA2

A one-step growth curve of isolated phage PSA2 was determined on SA33 according to the previously described method [55]. In summary, bacterial host SA33 was inoculated into 5 mL of LB broth and incubated at 37 °C for about 4 h to reach a concentration of approximately 10^8^ CFU/mL. One milliliter of bacterial culture was withdrawn and mixed with 1 mL of phage suspension to obtain an MOI of 0.01 and incubated for 10 min at 37 °C to allow the attachment of phage particles to the bacterial host cells. The mixture was then centrifuged at 10,000× *g* for 30 s at room temperature to remove unattached phage particles and to produce a pellet, which was then resuspended in 10 mL of fresh LB broth. The suspension was incubated in a shaking water bath at 37 °C. Samples (100 µL) were taken at 5 min intervals to examine phage titers by double-layer agar assay as mentioned above.

#### 4.5.3. Stability of Isolated Phage PSA2

The stability of phage PSA2 in different temperatures, pH, and NaCl conditions was determined following the previously described method [25,27]. The heat tolerance of phage PSA2 was evaluated by adding 100 µL of phage suspension (5 × 10^10^ PFU/mL) into 5 mL of preheated LB broth and incubated for 30 min in a shaking water bath at temperature conditions ranging from 40 °C to 90 °C. To examine the pH stability of phage PSA2, 100 µL of phage suspension (5 × 10^10^ PFU/mL) was inoculated into 5 mL of LB broth, pre-adjusted to pH values from 2 to 13, and then incubated at 37 °C for 60 min. The effect of NaCl on the viability of phage was assessed by adding 100 µL of phage suspension (5 × 10^10^ PFU/mL) to 5 mL of NaCl solution at various concentrations (1–11%) and incubated at 37 °C for 60 min. After incubation at different heat, pH, and NaCl conditions, phage titer was determined using double layer agar method as mentioned above.

#### 4.5.4. Evaluation of the Effect of Phage PSA2 on the Viability of MRSA SA33 in Raw Milk

The efficacy of phage PAS2 in controlling SA33 was investigated in raw milk following the previously described method [25]. Briefly, an overnight culture (100 µL) of SA33 was inoculated into 5 mL of raw milk to achieve a final concentration of approximately 10^5^ CFU/mL. The contaminated milk was then treated with 100 μL of isolated phages suspension (5 × 10^10^ PFU/mL) and incubated at 4 °C and 24 °C. At 2, 4, 6, and 24 h, a portion of the sample (100 μL) was serially diluted in PBS buffer before plating on TSA supplemented with cefoxitin at 8 mg/L. The plates were incubated at 37 °C to determine the viable counts of SA33.

### 4.6. Statistical Analysis

The experiments in this study were carried out repeatedly, at least three times. The values are presented as mean values and standard deviation of the mean. The differences between treatment and control groups were analyzed by *t*-test (Microsoft Excel 2019 for Mac OS).

## 5. Conclusions

This is the first report describing the isolation and application of phages in controlling enterotoxigenic MRSA in raw milk. In this study, *S. aureus* isolates were highly resistant to penicillin, erythromycin, and tetracycline. MRSA isolates displayed higher antibiotic resistance levels compared to MSSA. One MRSA isolate was found to carry the *sea* gene. The application of phage PSA2 significantly reduced SA33 in raw milk at both room temperature and refrigeration temperature. Phage PAS2 is a promising tool for controlling enterotoxigenic MRSA in raw milk.

## Figures and Tables

**Table 1:** Antibiotic resistance profile of *S. aureus* isolates.

Antibiotic Group	Antibiotic	No. Resistant Isolates (*n* = 48)	Resistance Rate (%)
Penicillins	penicillin	35	72.92
Cephalosporins	cefoxitin	6	12.50
Aminoglycosides	gentamicin	12	25.00
Tetracyclines	tetracycline	19	39.58
Phenicols	chloramphenicol	10	20.83
Macrolides	erythromycin	21	43.75
Lincosamides	clindamycin	9	18.75
Fluoroquinolones	ciprofloxacin	8	16.67
Sulfonamides	trimethoprim/sulfamethoxazole	13	27.08

**Table 2:** Antibiotic resistance patterns of *S. aureus* isolates.

No. of Antibiotics	Antibiotic Resistance Phenotype	No. of Resistance Isolates	Rate (%)
0	-	2	4.17
1	-PEN	13	27.08
1	-TET	2	4.17
1	-SXT	2	4.17
1	-ERY	3	6.25
2	-PEN-ERY	3	6.25
2	-PEN-TET	4	8.33
2	-PEN-GEN	1	2.08
2	-PEN-SXT	1	2.08
3	-TET-CHL-SXT	1	2.08
3	-ERY-CIP-SXT	1	2.08
4	-CHL-ERY-CLI-CIP	1	2.08
4	-GEN-ERY-CIP-SXT	1	2.08
4	-PEN-TET-ERY-SXT	1	2.08
4	-PEN-GEN-TET-CHL	1	2.08
5	-PEN-TET-CHL-ERY-SXT	1	2.08
5	-PEN-FOX-TET-ERY-SXT	1	2.08
6	-PEN-GEN-TET-CHL-ERY-SXT	1	2.08
6	-PEN-GEN-CHL-ERY-CLI-CIP	1	2.08
6	-PEN-GEN-TET-ERY-CLI-SXT	1	2.08
6	-PEN-FOX-GEN-TET-ERY-CLI	1	2.08
7	-PEN-GEN-TET-CHL-ERY-CLI-CIP	1	2.08
7	-PEN-FOX-GEN-TET-ERY-CLI-SXT	1	2.08
8	-PEN-FOX-GEN-TET-CHL-ERY-CLI-CIP	2	4.17
9	-PEN-FOX-GEN-TET-CHL-ERY-CLI-CIP-SXT	1	2.08
Resistant ≥ 1		46	95.83
MDR		17	35.42

penicillin: PEN; cefoxitin: FOX; gentamicin: GEN; tetracycline: TET; chloramphenicol: CHL; erythromycin: ERY; clindamycin: CLI; ciprofloxacin: CIP; trimethoprim/sulfamethoxazole: SXT.

**Table 3:** Molecular characteristics of methicillin-resistant *S. aureus* isolates.

Isolate ID	Antibiotic Resistance and Virulence-Associated Genes					Resistance Pattern
	*spa*	*mecA*	*mecC*	*pvl*	*se*	
SA6	*+*	*+*	−	−	−	PEN-FOX-GEN-TET-ERY-CLI
SA11	*+*	*+*	−	−	−	PEN-FOX-GEN-TET-CHL-ERY-CLI-CIP
SA14	*+*	*+*	−	−	−	PEN-FOX-GEN-TET-ERY-CLI-SXT
SA19	*+*	*+*	−	−	−	PEN-FOX-TET-ERY-SXT
SA33	*+*	*+*	−	−	*sea*	PEN-FOX-GEN-TET-CHL-ERY-CLI-CIP
SA45	*+*	*+*	−	−	−	PEN-FOX-GEN-TET-CHL-ERY-CLI-CIP-SXT

(+), Positive; (−), Negative; penicillin: PEN; cefoxitin: FOX; gentamicin: GEN; tetracycline: TET; chloramphenicol: CHL; erythromycin: ERY; clindamycin: CLI; ciprofloxacin: CIP; trimethoprim/sulfamethoxazole: SXT.

**Table 4:** Host range of isolated phages against MRSA isolates.

MRSA Isolates	PSA1	PSA2	PSA3	PSA4	PSA5
SA6	++	++	++	++	++
SA11	++	++	++	++	++
SA14	+	++	+	++	+
SA19	+	++	++	+	−
SA33	++	++	+	+	++
SA45	++	++	++	++	++
Total infected strains	6 (100%)	6 (100%)	6 (100%)	6 (100%)	83.33% (5/6)

− no lysis zone; + turbid zone; ++ clear lysis zone.

**Table 5:** Primer used for the detection of *mecA*, *mecC*, *spa*, *pvl*, and enterotoxin genes.

Gene	Oligonucleotide Sequence (5′–3′)	Product Size (bp)	Reference
*spa*	F-TAAAGACGATCCTTCGGTGAGC R-CAGCAGTAGTGCCGTTTGCTT	180–600	[61]
*mecA*	F-TCCAGATTACAACTTCACCAGG R-CCACTTCATATCTTGTAACG	162	
*mecC*	F-GAAAAAAAGGCTTAGAACGCCTC R-GAAGATCTTTTCCGTTTTCAGC	138	
*pvl*	F-GCTGGACAAAACTTCTTGGAATAT R-GATAGGACACCAATAAATTCTGGATTG	85	
*sea*	F-GCAGGGAACAGCTTTAGGC R-GTTCTGTAGAAGTATGAAACACG	520	[62]
*seb*	F-ACATGTAATTTTGATATTCGCACTG R-TGCAGGCATCATGTCATACCA	667	
*sec*	F-CTTGTATGTATGGAGGAATAACAA R-TGCAGGCATCATATCATACCA	284	
*sed*	F-GTGGTGAAATAGATAGGACTGC R-ATATGAAGGTGCTCTGTGG	171	
*see*	F-TACCAATTAACTTGTGGATAGAC R-CTCTTTGCACCTTACCGC	385	
*seg*	F-AAGTAGACATTTTTGGCGTTCC R-AGAACCATCAAACTCGTATAGC	287	
*seh*	F-CAACTGCTGATTTAGCTCAG R-GTCGAATGAGTAATCTCTAGG	359	
*sei*	F-CAACTCGAATTTTCAACAGGTACC R-CAGGCAGTCCATCTCCTG	466	

## Data Availability

The data that support the findings of this study are available from the corresponding author upon reasonable request.

## References

[1] Mora-Hernández Y., Vera Murguía E., Stinenbosch J., Hernández Jauregui P., van Dijl J.M., Buist G. (2021). Molecular typing and antimicrobial resistance profiling of 33 mastitis-related Staphylococcus aureus isolates from cows in the Comarca Lagunera region of Mexico. Sci. Rep..

[2] Sordillo L.M., Streicher K.L. (2002). Mammary Gland Immunity and Mastitis Susceptibility. J. Mammary Gland. Biol. Neoplasia.

[3] Lubna, Hussain T., Shami A., Rafiq N., Khan S., Kabir M., Khan N.U., Khattak I., Kamal M., Usman T. (2023). Antimicrobial Usage and Detection of Multidrug-Resistant *Staphylococcus aureus*: Methicillin- and Tetracycline-Resistant Strains in Raw Milk of Lactating Dairy Cattle. Antibiotics.

[4] Kovačević Z., Mihajlović J., Mugoša S., Horvat O., Tomanić D., Kladar N., Samardžija M. (2023). Pharmacoeconomic Analysis of the Different Therapeutic Approaches in Control of Bovine Mastitis: Phytotherapy and Antimicrobial Treatment. Antibiotics.

[5] Pedersen R.R., Krömker V., Bjarnsholt T., Dahl-Pedersen K., Buhl R., Jørgensen E. (2021). Biofilm Research in Bovine Mastitis. Front. Vet. Sci..

[6] Ararsa D. (2018). Isolation and Identification of Staphylococcus aureus from Dairy Farms in Bishoftu Town, Ethiopia. Juniper Online J. Public Health.

[7] Sarker S.C., Parvin M.S., Rahman A.K.M.A., Islam T. (2013). Prevalence and risk factors of subclinical mastitis in lactating dairy cows in north and south regions of Bangladesh. Trop. Anim. Health Prod..

[8] Kateete D.P., Kabugo U., Baluku H., Nyakarahuka L., Kyobe S., Okee M., Najjuka C.F., Joloba M.L. (2013). Prevalence and Antimicrobial Susceptibility Patterns of Bacteria from Milkmen and Cows with Clinical Mastitis in and around Kampala, Uganda. PLoS ONE.

[9] Basanisi M., La Bella G., Nobili G., Franconieri I., La Salandra G. (2017). Genotyping of methicillin-resistant Staphylococcus aureus (MRSA) isolated from milk and dairy products in South Italy. Food Microbiol..

[10] Schmidt T., Kock M.M., Ehlers M.M. (2017). Molecular Characterization of Staphylococcus aureus Isolated from Bovine Mastitis and Close Human Contacts in South African Dairy Herds: Genetic Diversity and Inter-Species Host Transmission. Front. Microbiol..

[11] Pexara A., Solomakos N., Sergelidis D., Govaris A. (2012). Fate of enterotoxigenic *Staphylococcus aureus* and staphylococcal enterotoxins in Feta and Galotyri cheeses. J. Dairy Res..

[12] De Buyser M.-L., Dufour B., Maire M., Lafarge V. (2001). Implication of milk and milk products in food-borne diseases in France and in different industrialised countries. Int. J. Food Microbiol..

[13] EFSA (2015). Scientific Opinion on the public health risks related to the consumption of raw drinking milk. EFSA J..

[14] Oliver S.P., Boor K.J., Murphy S.C., Murinda S.E. (2009). Food Safety Hazards Associated with Consumption of Raw Milk. Foodborne Pathog. Dis..

[15] Potter M.E., Kaufmann A.F., Blake P.A., Feldman R.A. (1984). Unpasteurized Milk: The Hazards of a Health Fetish. JAMA J. Am. Med. Assoc..

[16] Jay-Russell M.T. (2010). Raw (Unpasteurized) Milk: Are Health-Conscious Consumers Making an Unhealthy Choice?. Clin. Infect. Dis..

[17] Wang X., Liu Q., Zhang H., Li X., Huang W., Fu Q., Li M. (2018). Molecular Characteristics of Community-Associated *Staphylococcus aureus* Isolates from Pediatric Patients with Bloodstream Infections between 2012 and 2017 in Shanghai, China. Front. Microbiol..

[18] Neelam, Jain V.K., Singh M., Joshi V.G., Chhabra R., Singh K., Rana Y.S. (2022). Virulence and antimicrobial resistance gene profiles of Staphylococcus aureus associated with clinical mastitis in cattle. PLoS ONE.

[19] de Jong A., El Garch F., Simjee S., Moyaert H., Rose M., Youala M., Siegwart E. (2018). Monitoring of antimicrobial susceptibility of udder pathogens recovered from cases of clinical mastitis in dairy cows across Europe: VetPath results. Vet. Microbiol..

[20] Oliver S.P., Murinda S.E., Jayarao B.M. (2011). Impact of Antibiotic Use in Adult Dairy Cows on Antimicrobial Resistance of Veterinary and Human Pathogens: A Comprehensive Review. Foodborne Pathog. Dis..

[21] Titouche Y., Akkou M., Houali K., Auvray F., Hennekinne J. (2022). Role of milk and milk products in the spread of methicillin-resistant *Staphylococcus aureus* in the dairy production chain. J. Food Sci..

[22] Wörmann M., Pech J., Reich F., Tenhagen B.-A., Wichmann-Schauer H., Lienen T. (2024). Growth of methicillin-resistant *Staphylococcus aureus* during raw milk soft cheese-production and the inhibitory effect of starter cultures. Food Microbiol..

[23] Kamal R.M., Bayoumi M.A., El Aal S.F.A. (2013). MRSA detection in raw milk, some dairy products and hands of dairy workers in Egypt, a mini-survey. Food Control.

[24] Zhang Z., Wang J., Wang H., Zhang L., Shang W., Li Z., Song L., Li T., Cheng M., Zhang C. (2023). Molecular Surveillance of MRSA in Raw Milk Provides Insight into MRSA Cross Species Evolution. Microbiol. Spectr..

[25] Duc H.M., Son H.M., Ngan P.H., Sato J., Masuda Y., Honjoh K.-I., Miyamoto T. (2020). Isolation and application of bacteriophages alone or in combination with nisin against planktonic and biofilm cells of *Staphylococcus aureus*. Appl. Microbiol. Biotechnol..

[26] Hamdi S., Rousseau G.M., Labrie S.J., Tremblay D.M., Kourda R.S., Slama K.B., Moineau S. (2017). Characterization of two polyvalent phages infecting Enterobacteriaceae. Sci. Rep..

[27] Duc H.M., Son H.M., Yi H.P.S., Sato J., Ngan P.H., Masuda Y., Honjoh K.-I., Miyamoto T. (2020). Isolation, characterization and application of a polyvalent phage capable of controlling Salmonella and *Escherichia coli* O157:H7 in different food matrices. Food Res. Int..

[28] García-Anaya M.C., Sepulveda D.R., Sáenz-Mendoza A.I., Rios-Velasco C., Zamudio-Flores P.B., Acosta-Muñiz C.H. (2020). Phages as biocontrol agents in dairy products. Trends Food Sci. Technol..

[29] Principi N., Silvestri E., Esposito S. (2019). Advantages and limitations of bacteriophages for the treatment of bacterial infections. Front. Pharmacol..

[30] Loc-Carrillo C., Abedon S.T. (2011). Pros and cons of phage therapy. Bacteriophage.

[31] Kiani A.K., Anpilogov K., Dautaj A., Marceddu G., Sonna W.N., Percio M., Dundar M., Beccari T., Bertelli M. (2020). Bacteriophages in food supplements obtained from natural sources. Acta Biomed..

[32] Chai Z., Wang J., Tao S., Mou H. (2014). Application of bacteriophage-borne enzyme combined with chlorine dioxide on controlling bacterial biofilm. LWT Food Sci. Technol..

[33] Shrestha A., Bhattarai R.K., Luitel H., Karki S., Basnet H.B. (2021). Prevalence of methicillin-resistant Staphylococcus aureus and pattern of antimicrobial resistance in mastitis milk of cattle in Chitwan, Nepal. BMC Vet. Res..

[34] Király J., Hajdučková V., Gregová G., Szabóová T., Pilipčinec E. (2024). Resistant *S. aureus* Isolates Capable of Producing Biofilm from the Milk of Dairy Cows with Subclinical Mastitis in Slovakia. Agriculture.

[35] Liu J., Wang X., Bi C., Mehmood K., Ali F., Qin J., Han Z. (2022). Molecular characterization of multi-drug-resistant Staphylococcus aureus in mastitis bovine milk from a dairy farm in Anhui, China. Front. Vet. Sci..

[36] Wang W., Lin X., Jiang T., Peng Z., Xu J., Yi L., Li F., Fanning S., Baloch Z. (2018). Prevalence and Characterization of *Staphylococcus aureus* Cultured from Raw Milk Taken from Dairy Cows with Mastitis in Beijing, China. Front. Microbiol..

[37] Ren Q., Liao G., Wu Z., Lv J., Chen W. (2020). Prevalence and characterization of Staphylococcus aureus isolates from subclinical bovine mastitis in southern Xinjiang, China. J. Dairy Sci..

[38] Salam A., Al-Amin Y., Salam M.T., Pawar J.S., Akhter N., Rabaan A.A., Alqumber M.A.A. (2023). Antimicrobial Resistance: A Growing Serious Threat for Global Public Health. Healthcare.

[39] Martin M.J., Thottathil S.E., Newman T.B. (2015). Antibiotics Overuse in Animal Agriculture: A Call to Action for Health Care Providers. Am. J. Public Health.

[40] Xu C., Kong L., Gao H., Cheng X., Wang X. (2022). A Review of Current Bacterial Resistance to Antibiotics in Food Animals. Front. Microbiol..

[41] Van T.T.H., Yidana Z., Smooker P.M., Coloe P.J. (2020). Antibiotic use in food animals worldwide, with a focus on Africa: Pluses and minuses. J. Glob. Antimicrob. Resist..

[42] Tiseo K., Huber L., Gilbert M., Robinson T.P., Van Boeckel T.P. (2020). Global Trends in Antimicrobial Use in Food Animals from 2017 to 2030. Antibiotics.

[43] Gao J., Ferreri M., Yu F., Liu X., Chen L., Su J., Han B. (2012). Molecular types and antibiotic resistance of *Staphylococcus aureus* isolates from bovine mastitis in a single herd in China. Vet. J..

[44] Virto M., Santamarina-García G., Amores G., Hernández I. (2022). Antibiotics in Dairy Production: Where Is the Problem?. Dairy.

[45] Wang H., Shen J., Zhu C., Ma K., Fang M., Li B., Wang W., Xue T. (2022). Antibiotics Resistance and Virulence of *Staphylococcus aureus* Isolates Isolated from Raw Milk from Handmade Dairy Retail Stores in Hefei City, China. Foods.

[46] Qolbaini E.N., Khoeri M.M., Salsabila K., Paramaiswari W.T., Tafroji W., Artika I.M., Safari D. (2021). Identification and antimicrobial susceptibility of methicillin-resistant *Staphylococcus aureus*-associated subclinical mastitis isolated from dairy cows in Bogor, Indonesia. Vet. World.

[47] Khazaie F., Ahmadi E. (2021). Bovine subclinical mantis-associated methicillin-resistant *Staphylococcus aureus*, selective genotyping and antimicrobial susceptibility profile of the isolates in Kurdistan province of Iran. Iran. J. Microbiol..

[48] Deurenberg R., Vink C., Kalenic S., Friedrich A., Bruggeman C., Stobberingh E. (2007). The molecular evolution of methicillin-resistant Staphylococcus aureus. Clin. Microbiol. Infect..

[49] Spanu V., Spanu C., Virdis S., Cossu F., Scarano C., De Santis E.P.L. (2012). Virulence factors and genetic variability of *Staphylococcus aureus* strains isolated from raw sheep’s milk cheese. Int. J. Food Microbiol..

[50] Ortega E., Abriouel H., Lucas R., Gálvez A. (2010). Multiple Roles of *Staphylococcus aureus* Enterotoxins: Pathogenicity, Superantigenic Activity, and Correlation to Antibiotic Resistance. Toxins.

[51] Monistero V., Graber H.U., Pollera C., Cremonesi P., Castiglioni B., Bottini E., Ceballos-Marquez A., Lasso-Rojas L., Kroemker V., Wente N. (2018). *Staphylococcus aureus* Isolates from Bovine Mastitis in Eight Countries: Genotypes, Detection of Genes Encoding Different Toxins and Other Virulence Genes. Toxins.

[52] Atterbury R.J. (2009). Bacteriophage biocontrol in animals and meat products. Microb. Biotechnol..

[53] Hagens S., Loessner M.J. (2010). Bacteriophage for Biocontrol of Foodborne Pathogens: Calculations and Considerations. Curr. Pharm. Biotechnol..

[54] Mohammadian F., Rahmani H.K., Bidarian B., Khoramian B. (2022). Isolation and evaluation of the efficacy of bacteriophages against multidrug-resistant (MDR), methicillin-resistant (MRSA) and biofilm-producing strains of *Staphylococcus aureus* recovered from bovine mastitis. BMC Vet. Res..

[55] Duc H.M., Son H.M., Honjoh K.-I., Miyamoto T. (2018). Isolation and application of bacteriophages to reduce Salmonella contamination in raw chicken meat. LWT.

[56] Galarce N.E., Bravo J.L., Robeson J.P., Borie C.F. (2014). Bacteriophage cocktail reduces Salmonella enterica serovar Enteritidis counts in raw and smoked salmon tissues. Rev. Argent. Microbiol..

[57] Hungaro H.M., Mendonça R.C.S., Gouvêa D.M., Vanetti M.C.D., de Oliveira Pinto C.L. (2013). Use of bacteriophages to reduce Salmonella in chicken skin in comparison with chemical agents. Food Res. Int..

[58] da Silva N., Taniwaki M.H., Junqueira V.C.A., Silveira N., Okazaki M.M., Romeiro Gomes R.A. (2012). Microbiological Examination Methods of Food and Water: A Laboratory Manual.

[59] Louie L., Goodfellow J., Mathieu P., Glatt A., Louie M., Simor A.E. (2002). Rapid Detection of Methicillin-Resistant Staphylococci from Blood Culture Bottles by Using a Multiplex PCR Assay. J. Clin. Microbiol..

[60] CLSI (2017). Performance Standards for Antimicrobial Susceptibility Testing.

[61] EURL-AR Protocol for PCR amplification of mecA, mecC (mecAlga251), spa and pvl. September 2012, pp. 1–5. https://www.eurl-ar.eu/CustomerData/Files/Folders/21-protocols/279_pcr-spa-pvl-meca-mecc-sept12.pdf.

[62] Savariraj W.R., Ravindran N.B., Kannan P., Paramasivam R., Senthilkumar T., Kumarasamy P., Rao V.A. (2019). Prevalence, antimicrobial susceptibility and virulence genes of *Staphylococcus aureus* isolated from pork meat in retail outlets in India. J. Food Saf..

[63] Minh D.H., Minh S.H., Honjoh K.-I., Miyamoto T. (2016). Isolation and bio-control of Extended Spectrum Beta-Lactamase (ESBL)-producing *Escherichia coli* contamination in raw chicken meat by using lytic bacteriophages. LWT Food Sci. Technol..

